# Labyrinthitis ossificans following severe acute otitis media

**DOI:** 10.1002/ccr3.5898

**Published:** 2022-05-24

**Authors:** Kensuke Uraguchi, Shin Kariya, Mizuo Ando

**Affiliations:** ^1^ Department of Otolaryngology‐Head and Neck Surgery Okayama University Graduate School of Medicine, Dentistry and Pharmaceutical Sciences Okayama Japan

**Keywords:** acute otitis media, labyrinthitis, labyrinthitis ossificans, sensorineural hearing loss

## Abstract

Labyrinthitis occurs because of the inflammation of the inner ear. We present a rare case of labyrinthitis ossificans following an acute otitis media. The T2‐weighted magnetic resonance imaging showed decreased signal intensity in the right inner ear due to labyrinthitis ossificans, consistent with the clinical presentation.

## Graphical abstract

Labyrinthitis is the inflammation of the inner ear and can result in labyrinthitis ossificans. The T2‐weighted magnetic resonance imaging can play a crucial role in the diagnosis of labyrinthitis ossificans.

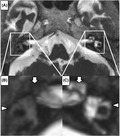

An 83‐year‐old woman without immunosuppression became aware of bilateral hearing loss. She later developed vertigo and was referred to our department. Clinical findings revealed bilateral acute otitis media (AOM) with the perforation of the tympanic membrane (Figure 1). Pure tone audiometry revealed severe sensorineural hearing loss (SNHL) in the right ear. A spontaneous left‐beating nystagmus appeared, and the patient could not stand by herself. She was hospitalized and given antibiotics. The culture test of the ear pus revealed *Streptococcus pyogenes*. Given her recovery from AOM, we administered corticosteroid. On the 30th day of the first visit, T2‐weighted magnetic resonance imaging (MRI) showed decreased signal intensity in the right inner ear (Figure 2). Her vertigo and nystagmus improved. Although the tympanic membrane healed, her SNHL in the right ear showed no improvement.

**FIGURE 1:**
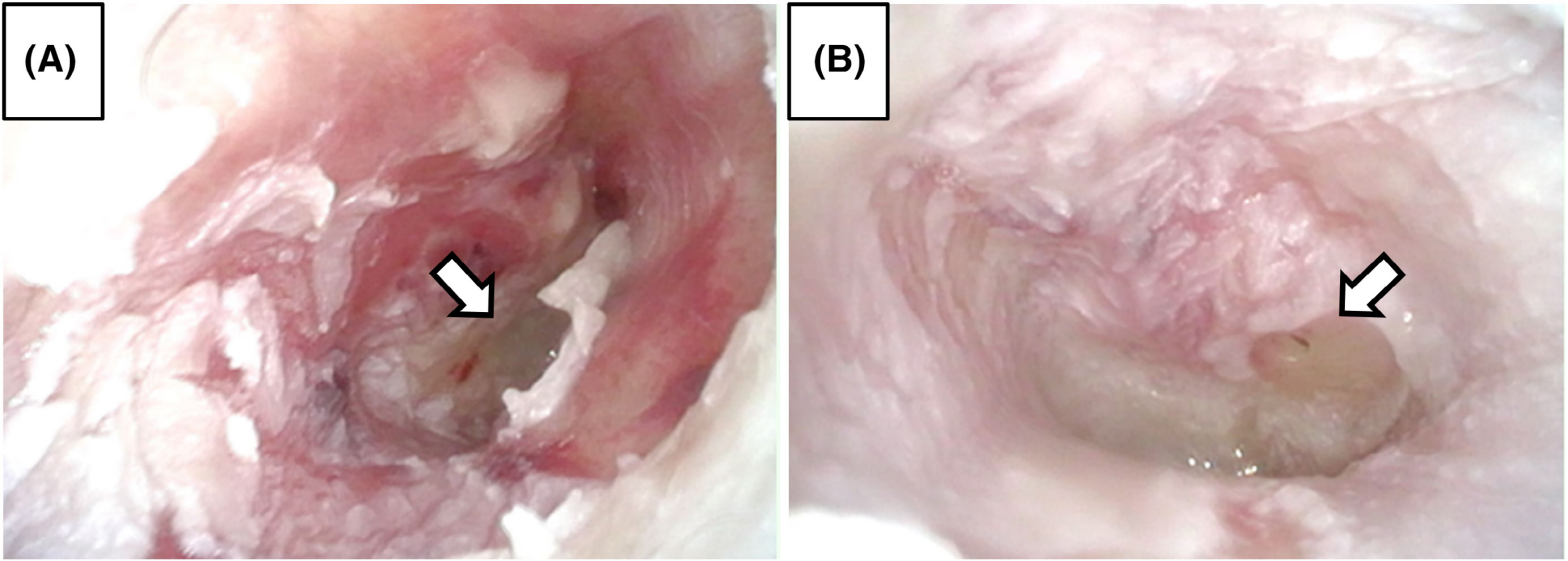
Endoscopic finding of tympanic membrane. (A) Right (B) left. Both had perforation of the tympanic membrane and pus discharge (arrow: perforation of tympanic membrane)

**FIGURE 2:**
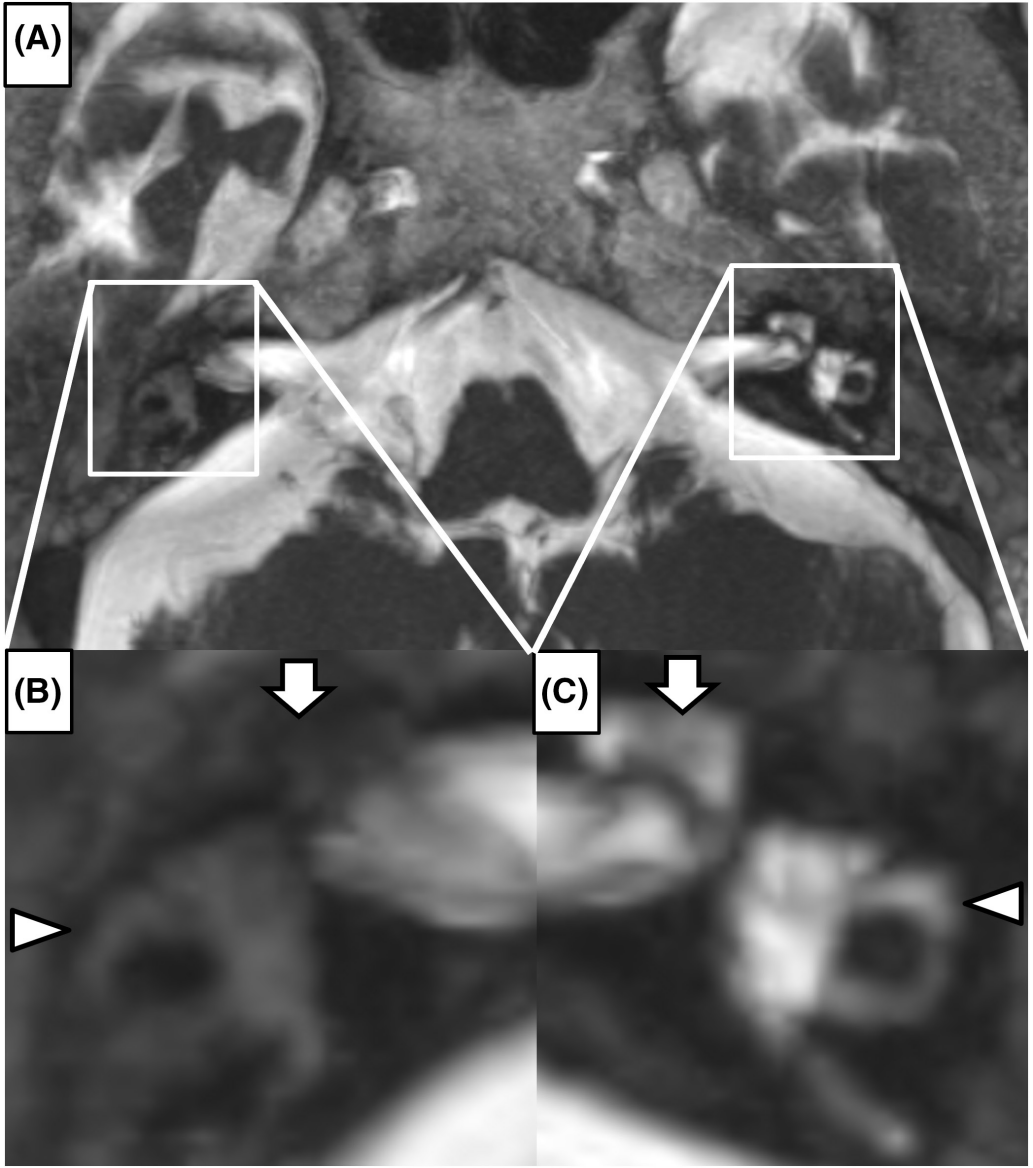
(A) MRI of T2‐weighted image showed decreased signal intensity in the right inner ear, demonstrating the process of inner ear ossification. (B) Detailed view of the right inner ear; (C) detailed view of the left inner ear (arrow: cochlear; arrowhead: semicircular canal)

Labyrinthitis can cause fibrosis in the membranous labyrinth filled with ossified material, resulting in labyrinthitis ossificans. In such cases, patients often present with irreversible SNHL and vertigo due to labyrinthitis ossification.^1^ The administration of corticosteroid can effectively prevent the development of labyrinthitis ossificans.^2^ T2‐weighted MRI can demonstrate decreased signal intensity in the inner ear because inner ear fibrosis or ossification decreases perilymphatic fluid.^1^

## AUTHOR CONTRIBUTIONS

KU cared for the patient, and contributed to the editing of the manuscript and preparation of the figure. SK and MA contributed to the design of the case report presentation, and performed the final revision of the manuscript.

## CONFLICTS OF INTEREST

The authors declare that they have no conflict of interest.

## ETHICAL APPROVAL

Institutional review board approval was deemed unnecessary.

## CONSENT

Written informed consent was obtained from the patient and patient's family for publication of this case report and accompanying images.

## Data Availability

The data and original images are available from the corresponding author upon reasonable request.
